# Circumcision

**DOI:** 10.4103/2589-0557.74967

**Published:** 2010

**Authors:** Poonam Puri, Joginder Kumar, V. Ramesh

**Affiliations:** Department of Dermatology, Venereology and Leprosy, Vardhman Mahavir Medical College and Safdarjung Hospital, New Delhi - 110 029, India

**Keywords:** Circumcision, genital mutilation, infibulation, phimosis

## Abstract

Circumcision is one of the oldest and the most controversial surgical procedures performed worldwide and is almost universal among Muslim and Jewish men. Most medical institutions in various countries agree that although there may be health benefits, there is no medical justification for routine circumcision in neonates or children. It should be performed only for established medical reasons and should not be universally recommended. There are modern techniques that provide safer, simpler, quicker, and cheaper alternatives to the traditional means of circumcision with good functional and cosmetic results. Female genital mutilation (FGM) includes procedure that alters or injures female genital organs for nonmedical reasons. Various degrees of FGM are prevalent, the most mutilating one being infibulation. There are numerous gynecologic and obstetrical complications with infibulation. FGM also plays a significant role in facilitating the transmission of HIV infection through numerous mechanisms. Health care providers have an important role to play in the eradication of this practice. Increased professional and public awareness about such a practice is required.

## MALE CIRCUMCISION

### Introduction

Circumcision is the removal of some or the entire foreskin (prepuce) from the penis.[1] The word “circumcision” comes from Latin circum (meaning “around”) and cædere (meaning “to cut”). In Judaism male circumcision is considered a commandment from God. Male circumcision is widely practiced and considered to be a Sunnah[2] in Islam. In Jews, it is performed without anesthesia on the child’s 8th day of life, whereas in Muslims between 4 and 13 years of age.[3]

### History

Circumcision is one of the oldest and most controversial surgical procedures.[4] It has been described in Egyptian papyri and wall carvings dating back to 4000 BC[5] where the ancient mummies were found to be circumcised.[3] Circumcision was probably started as a public health measure for preventing recurrent balanitis due to accumulation of sand under the foreskin in the ancient societies of the Middle East.[6]

### Prevalence

World Health Organization (WHO) estimates that 30% of all males in the world 15 years and older are circumcised and almost 70% of them are Muslims.[7]

### Modern circumcision procedures

The procedure is done under local anesthesia using either topical local anesthetic cream, which is applied 60–90 min prior to the procedure at the site of circumcision, or a local anesthetic is infiltrated at the base of the penis to block the dorsal penile nerve or injected around the middle of the penis as a subcutaneous ring block.[8] For infant circumcision, clamps along with a restraining device are used.[9] First, the extent of the foreskin to be removed is determined. The foreskin is then opened via the preputial orifice to reveal the glans underneath. The inner lining of the foreskin (preputial epithelium) is then bluntly separated from its attachment to the glans. The restraining device is then placed till blood flow has stopped. Finally the foreskin is amputated.[8] Adult circumcisions are often performed without clamps and require 4–6 weeks of abstinence from masturbation or intercourse after the operation to allow the wound to heal.[9] The most common complications are blood loss and infection. The long-term complication of circumcision is meatal stenosis, which can lead to dysuria, incontinence, bleeding after urination, and urinary tract infections.[10] Other complications include concealed penis,[11] urinary fistulas, chordee, cysts, lymphedema, ulceration of the glans, hypospadias, epispadias, and impotence. Most of these complications are preventable as these occur by untrained persons who are neither urologists nor surgeons. Occasional death estimated at 1 per 500,000 circumcisions has been cited by American Academy of Family Physicians.[12]

### Ethical issues

Controversy exists in new born males regarding indications of ritual circumcision on religious groundsBecause neonatal circumcision is not necessary for the child’s wellbeing, various medical associations, such as the American Academy of Pediatrics (AAP), British Medical Associations and Canadian Paediatrics Society, Royal Australasian College of Physicians, suggested that it should be performed only for established medical reasons and not to be universally recommended.[13] Infant circumcision infringes upon individual autonomy and represents a violation of human rights.[14] Parents who believe that circumcision is of medical benefit should be educated on the pros and cons of the procedure, and available alternatives to circumcision. They should be explained that their child can lead a healthy life even with an intact foreskin. Such information enables them to make the right choice for their child. UNAIDS states that “male circumcision is a voluntary surgical procedure and health care providers must ensure that men and young boys are provided information to enable them to make decision either for or against getting circumcised.”

### Routine neonatal circumcision

Routine neonatal circumcision has advantages as well as drawbacks. The beneficial effects include prevention of phimosis, paraphimosis, and balanoposthitis. The risk of urinary tract infection has been shown to decrease from 7 per 1000 to 2 per 1000 after circumcision.[6] Few studies show that human papillomavirus infection of penis is less in circumcised males and their female partners have less chances of developing cervical cancer compared with their uncircumcised counterparts.[15] The incidence of sexually transmitted diseases is 10% lower in circumcised males.[6] There is clear evidence that circumcised men are at a significantly lower risk of acquiring HIV infection.[16] The disadvantage with routine neonatal male circumcision is that it is frequently performed by untrained persons without anesthesia. The babies experience pain more intensely for a prolonged period and over a wider area of the body than older children do.[5] The foreskin not only protects the glans penis but has specialized nerve endings (Meissner corpuscles) for fine touch, which enhances sexual pleasure. Hence, ritual circumcision removes the erogenous tissue, which serves important protective, sensory, and sexual purposes.[13]

### Sexually transmitted diseases and circumcision

There are conflicting reports on the effect of circumcision on the incidence of sexually transmitted infections (STIs). A meta-analysis from —26 studies found that circumcision was associated with lower rates of syphilis, chancroid, and genital herpes.[17] Circumcision may protect against both bacterial and viral STIs because the warm, moist area under the foreskin provides a suitable location for the pathogens to replicate. In addition, uncircumcised men may be at increased risk due to entry of pathogens through the inner surface of the foreskin and frenulum. The physical location of ulcers may also affect the role of circumcision on genital ulcerative diseases. Chancroid frequently occurs on the external and internal surfaces of the foreskin,[18] hence circumcision may be more protective against chancroid than against syphilis and herpes, where lesions tend to be found more on the male genitalia. Potential male circumcision interventions to reduce HIV in high-risk populations may provide additional benefit by protecting against other STIs. Three randomized controlled trials in sub-Saharan Africa have shown that circumcision may be protective against genital ulcer disease, herpes simplex type 2, trichomonas vaginalis, and human papillomavirus infection in men.[19] However, a large randomized prospective trial in Uganda found a reduction in herpes simplex virus type 2 (HSV-2) infection, but not syphilis infection in the circumcised patients.[20] A prospective trial in India found that circumcision offered no protective benefit against HSV-2, syphilis, or gonorrhea.[21] A study of 5925 women from Uganda, Zimbabwe, and Thai described that the circumcision status of their partner did not significantly affect the incidence of Chlamydia, gonorrhea, or trichomoniasis.[22]

### Circumcision and HIV

There is clear evidence that circumcised men are at a significantly lower risk of acquiring HIV infection,[13–15] probably because the inner surface of the foreskin contains numerous Langerhans cells and CD4+ T lymphocytes (primary HIV-1 target cells),[21] and the warm, moist environment under the foreskin.[23] The latter could also facilitate infection with other sexually transmitted pathogens The protective effect of circumcision on HIV is especially strong among populations more highly exposed to STIs, suggesting that part of the effect on HIV may be mediated via protection against other STIs that facilitate HIV transmission.[13] This explains the surprising low rates of HIV in Islamic nations, such as Egypt, Sudan, Iran, Iraq, Pakistan, Bangladesh, and Indonesia, compared with their neighbors.[15] The WHO (2007), the Joint United Nations Programme on HIV/AIDS (UNAIDS; 2007), and the Centers for Disease Control and Prevention (CDC; 2008) state that male circumcision significantly reduces the risk of HIV acquisition by men during penile–vaginal sex, but is not a substitute for other interventions to prevent HIV transmission.[24,25]

### Sexual effects of circumcision

Circumcision increases the ejaculatory latency time without any adverse effect on sexual functions.[26] This may be considered an advantage rather than a complication.

### Medical indications of circumcision in children

Circumcision is recommended for acquired phimosis, paraphimosis, recurrent balanitis, and recurrent urinary tract infections.[27]

### Contraindications

Circumcision should not be performed in children with congenital penile anomalies, such as hypospadias, epispadias, chordee, megalourethra, or webbed penis. It should be avoided in patients with bleeding disorders.

### The alternatives to radical circumcision

Radical circumcision is now an obsolete procedure. It is a painful procedure with prolonged recovery time. There is destruction of functional tissue sometimes associated with serious complications. The alternative conservative treatment for relieving preputial stenosis consists of topical steroid application (0.05% clobetasol propionate cream applied twice daily for 1–2 months.[28] Gentle stretching of the foreskin over a period of time will open up the narrow preputial opening, while preserving the foreskin. Preputioplasty and dorsal slit are conservative alternatives to the radical circumcision.[29] They are rapid and less painful procedures with early recovery. “Triple incision preputioplasty” is a simple, safe, and rapid technique with good functional and cosmetic outcome. In this procedure, 3 longitudinal incisions on the stenosing segment are made, which are sutured diagonally.[30] Nd:YAG laser contact technique[31] and octyl cyanoacrylate glue[32] are other modalities used to perform sutureless circumcision.

### Key facts

1. Circumcision should be performed only for established medical reasons and should not be recommended routinely.
2. The health benefits should be weighed against the potential risks that the procedure offers.

## FEMALE CIRCUMCISION

### Introduction

Female circumcision is a procedure involving partial or total removal of the external genitalia for cultural, religious, or nontherapeutic reasons.[33] It is mostly carried out on young girls between infancy to adolescence. The procedure has no health benefits for girls and women. The WHO recommends the use of the term female genital mutilation (FGM).[34]

### History

FGM has been practiced for centuries. Egyptian mummies were found to have been circumcised as far back as 200 BC In the 19th Century it was practiced in Europe and North America as a remedy for ailments, such as epilepsy, hysteria, and masturbation.[35]

### Prevalence

An estimated 130 million girls and women worldwide have been subjected to some form of FGM, with over 3 million girls at risk of undergoing FGM every year. FGM is mainly practiced in 28 countries of Africa. The country where FGM is most prevalent is Egypt, followed by Sudan, Ethiopia, and Mali. The practice can also be found among some ethnic groups in South America. Immigration has led to its spread in Europe, Australia, and the United States. Daughters of some tradition-minded families undergo FGM while visiting their home countries on vacation.

### Procedure

The WHO divides the procedure into 4 major types[36] :

#### Type 1

Clitoridectomy: partial or total removal of the clitoris (clitoridectomy) and/or the prepuce (clitorial hood). It is similar to male circumcision. It is termed Sunnah for men and Makramah for women. Makramah means honorable deed.[37]

#### Type 2

Excision: partial or total removal of the clitoris and the labia minora, with or without excision of the labia majora.

#### Type 3

Infibulation: In this procedure, there is narrowing of the vaginal orifice with creation of a covering seal by cutting and repositioning the labia minora and/or the labia majora, with or without excision of the clitoris. There is extensive tissue removal of the external genitalia, including the entire labia minora and the inside of the labia majora. Only an opening at the inferior portion of the vulva remains to allow urine and menstrual blood to pass through.[38] Generally, a local village practitioner or a midwife of the village performs infibulation in the bush, without asepsis or anesthesia. The term Infibulation refers to the use of clasp (infibula) to keep the cut edges of the vagina together. It accounts for 10% of all FGM procedures from Africa.[39] Reversal of infibulation (anterior episiotomy) is possible to allow sexual intercourse or during labor to allow vaginal delivery, so the infibulation is opened completely and may be closed after delivery.

#### Type 4

Other types: All other harmful procedures to the female genitalia for nonmedical purposes, for example, pricking the clitoris with needles, piercing, incising, scraping, and burning of the genital area.[36]

### Consequences and complications

Complications of female circumcision may occur immediately after the procedure or later and these can be physical or psychological. They occur most frequently with the procedure of infibulation.[40] Immediate complications include severe pain, shock due to excessive bleeding, tetanus, or sepsis, open sores in the genital region, injury to the adjacent genital tissue, and urine retention. Long-term consequences include recurrent bladder and urinary tract infections, cysts, infertility, pelvic inflammatory disease, and a higher risk of childbirth complications and newborn deaths. Women who have been infibulated face a lot of difficulty during delivery, especially if the infibulation is not opened completely. There are chances of severe tearing of the infibulated area or fetal death.[41]

### Cultural, religious, and social causes

The FGM is performed as a result of cultural, religious, and social factors within families and communities. FGM is considered a necessary part of raising a girl properly and to prepare her for adulthood and marriage. FGM is said to ensure a “proper” sexual behavior, premarital virginity, and marital fidelity, and reduced woman’s libido.[42] Certain cultural ideas of femininity and modesty include the notion that girls are “clean” and “beautiful” after removal of body parts that are considered “male” or “unclean.” A few religious leaders still promote FGM, some consider it irrelevant to religion while others contribute to its elimination.

### Circumcision and HIV

A few recent reviews have suggested that the female genital circumcision may increase the risk of HIV.[43] The various proposed mechanisms include nonsterile procedures, an increased number of blood transfusions due to blood loss during surgery, increased anal intercourse due to difficult or painful vaginal intercourse, and tearing of the vagina during intercourse. Other studies have found no statistically significant associations.[44]

### Circumcision and sexual effects

The effect of FGM on a woman’s sexual experience varies depending on many factors. Clitoris stimulation is not solely responsible for the sexual excitement as sexual arousal during intercourse involves stimulation of complex series of nerve endings in and around vagina, labia minora and majora, cervix, uterus, and clitoris in addition to psychological response and mindset.[45] FGM does not eliminate sexual pleasure totally for every woman who undergoes the procedure, but it does reduce the likelihood of orgasm.

### To end the practice

Despite laws forbidding the practice, FGM still remains a tradition in many societies and cultural groups. Few countries that prohibited FGM still experience the practice in secrecy. In many cases, enforcement of this prohibition is a low priority for the governments. Other countries have tried to educate practitioners to make it easier and safer instead of outlawing the practice entirely. However, with pressure from the WHO and other groups, laws are being passed in regard to FGM. On June 28, 2007 Egypt banned female genital circumcision after the death of 12-year-old Badour Shaker during a genital circumcision. The United Nations Population Fund (UNFPA) has declared February 6 as the International Day against FGM.

### Key facts

1. It has no health benefits for girls and women.
2. FGM is considered as infringement of human rights against girls and women who are unable to give informed consent.

## Multiple Choice Questions

Q.1. Which of the following is homologous to glans penis?

- Labia majora
- Labia minora
- Mons pubis
- Clitoris

Q.2. Which one of the following statements is correct?

- Circumcising newborn boys is necessary for their sexual health
- There are no valid medical indications to recommend the routine circumcision of newborn boys
- Circumcising newborn boys is detrimental to their health
- Circumcising newborn boys often results in life-threatening health complications

Q.3. Type 3 FGM involves

- Excision of the prepuce only
- Excision of part or all of the external genitalia
- Burning the clitoris
- Introducing corrosive substance in the vagina

Q.4. Decreased incidence of HIV in circumcised cases is due to

- Poor hygiene
- Decreased sexual activity
- Decrease in the number of Langerhans cells
- Presence of commensals in the preputial sac

Q.5. Find the incorrect statement about male circumcision

- Male circumcision is associated with a lower risk of HIV infection
- It reduces risk of some STIs
- It causes infertility
- It should not be performed routinely but for established medical reasons

### Answers

Q.1. d. Clitoris
Q.2. b. There are no valid medical indications to recommend the routine circumcision of newborn boys
Q.3. b. Excision of part or all of the external genitalia
Q.4. c. Decrease in the number of Langerhans cells
Q.5. c. It causes infertility

## References

[1] (1913). Webster’s Revised Unabridged Dictionary.

[2] Rizvi SA, Naqvi SA, Hussain M, Hasan AS (1999). Religious circumcision: A Muslim view. BJU Int.

[3] Williams N, Kapila L (1993). Complications of circumcision. Br J Surg.

[4] Alanis MC, Lucidi RS (2004). Neonatal circumcision: A review of the world’s oldest and most controversial operation. Obstet Gynecol Surv.

[5] Goodman J (1999). Jewish circumcision: An alternative perspective. BJU Int.

[6] Hutson JM (2004). Circumcision: A surgeon’s perspective. J Med Ethics.

[7] (2007). Male circumcision: Global trends and determinants of prevalence, safety and acceptability.

[8] Circumcision policy statement (1999). American Academy of Pediatrics. Task Force on Circumcision. Pediatrics.

[9] Holman JR, Lewis EL, Ringler RL (1995). Neonatal circumcision techniques. Am Fam Physician.

[10] Van Howe RS (2006). Incidence of meatal stenosis following neonatal circumcision in a primary care setting. Clin Pediatr (Phila).

[11] Trier WC, Drach GW (1973). Concealed Penis. Another Complication of Circumcision. Am J Dis child.

[12] (2007). Paediatric Death Review Committee: Office of the Chief Coroner of Ontario. Coroner’s Corner Circumcision: A minor procedure?”. Paediatr Child Health.

[13] Viens AM (2004). Value judgment, harm, and religious liberty. J Med Ethics.

[14] Tanne HJ (2005). US group lobbies UN to outlaw male circumcision”. Br Med J.

[15] Short RV (2004). Male circumcision: A scientific perspective. J Med Ethics.

[16] Gray RH, Kigozi G, Serwadda D, Makumbi F, Watya S, Nalugoda F (2007). Male circumcision for HIV prevention in men in Rakai, Uganda: A randomised trial. Lancet.

[17] Weiss HA, Thomas Sl, Munabi SK, Hayes RJ (2006). Male circumcision and risk of syphilis, chancroid, and genital herpes: A systematic review and meta-analysis. Sex Transm Infect.

[18] Nsanze H, Fast MV, D’Costa LJ, Tukei P, Curran J, Ronald A (1981). Genital ulcers in Kenya. Clinical and laboratory study. Br J Vener Dis.

[19] Larke N (2010). Male circumcision, HIV and sexually transmitted infections: A review. Br J Nurs.

[20] Tobian AA, Serwadda D, Quinn TC, Kigozi G, Gravitt PE, Laeyendecker O (2009). Male circumcision for the prevention of HSV-2 and HPV infections and syphilis. N Engl J Med.

[21] Reynolds SJ, Shepherd ME, Risbud AR, Gangakhedkar RR, Brookmeyer RS, Divekar AD (2004). Male circumcision and risk of HIV-1 and other sexually transmitted infections in India. Lancet.

[22] Turner AN, Morrison CS, Padian NS, Kaufman JS, Behets FM, Salata RA (2008). Male circumcision and women’s risk of incident chlamydial, gonococcal, and trichomonal infections. Sex Transm Dis.

[23] Szabo R, Short RV (2000). How does male circumcision protect against HIV infection?. BMJ.

[24] (2007). New Data on Male Circumcision and HIV Prevention: Policy and Programme Implications.

[25] (2008). Male Circumcision and Risk for HIV Transmission and Other Health Conditions: Implications for the United States.

[26] Senkul T, Iserl C, Sen B, Karademir K, Saracoglu F, Erden D (2004). Circumcision in adults: effects on sexual functions. Urol.

[27] Cantu S Circumcision. e Medicine Journal [serial online].

[28] Jorgensen ET, Svensson A (1993). The treatment of phimosis in boys with a potent topical steroid (0.05% clobetasol propionate) cream. Acta Derm Venereol.

[29] Cuckow PM, Rix G, Mouriquand PD (1994). Preputial plasty: A good alternative to circumcision. J Paediatr Surg.

[30] Pascotto R, Giancotti E (1998). The treatment of phimosis in the childhood without circumcision: Plastic repair of the prepuce. Minerva Chir.

[31] Vaos G (2004). Circumcision with the Nd: YAG laser contact technique compared with conventional surgery. Photomed Laser Surg.

[32] Subramaniam R, Jacobsen AS (2004). Sutureless circumcision: A prospective randomised controlled study. Paed Surg Int.

[33] Definition used by the World Health Organization.

[34] Schoen EJ (1995). Female circumcision(letter). N Engl J Med.

[35] Dirie MA, Lindmark G (1992). The risk of medical complications after female circumcision. East Afr Med J.

[36] (2008). Eliminating female Genital Mutilation An interagency statement OHCHR, UNAIDS, UNFPA, UNHCR, UNICEF, UNIFEM, WHO Department of Reproductive Health and Research (RHR).

[37] Abu Daia JM (2000). Female circumcision. Saudi Med J.

[38] Verzin JA (1975). Sequelae of female circumcision. Trop Doct.

[39] (2000). Female genital mutilation.

[40] Nicoletti A (2007). Female Genital Cutting. J Pediatr Adolesc Gynecol.

[41] Toubia N (1994). Female Circumcision as a Public Health Issue. N Engl J Med.

[42] Braddy CM, Files JA (2007). Female genital mutilation: Cultural awareness and clinical considerations. J Midwifery Womens Health.

[43] Monjok E, Essien EJ, Holmes L (2007). Female genital mutilation: Potential for HIV transmission in sub-Saharan Africa and prospect for epidemiologic investigation and intervention. Afr J Reprod Health.

[44] Mboto CI, Fielder M, Davies Russell A, Jewell AP (2009). Prevalence of HIV-1, HIV-2, hepatitis C and co-infection in the Gambia. West Afr J Med.

[45] Mah K, Binik YM (2005). Are orgasms in the mind of the body. Psychosocial versus physiological correlates of orgasmic pleasure and satisfaction?. J Sex Marital Ther.

